# Integrated single-cell and bulk RNA sequencing analysis identifies a neoadjuvant chemotherapy-related gene signature for predicting survival and therapy in breast cancer

**DOI:** 10.1186/s12920-023-01727-0

**Published:** 2023-11-23

**Authors:** Xiaojun Zhang, Ran Feng, Junbin Guo, Lihui Pan, Yarong Yao, Jinnan Gao

**Affiliations:** 1 General Surgery Department, Third Hospital of Shanxi Medical University, Taiyuan, Shanxi 030032 China; 2 Yangquan Coal Industry (Group) General Hospital, Yangquan, Shanxi 045008 China

**Keywords:** Breast cancer, Neoadjuvant chemotherapy, Prognostic model, Multi-omics integration

## Abstract

Neoadjuvant chemotherapy (NAC) is a well-established treatment modality for locally advanced breast cancer (BC). However, it can also result in severe toxicities while controlling tumors. Therefore, reliable predictive biomarkers are urgently needed to objectively and accurately predict NAC response. In this study, we integrated single-cell and bulk RNA-seq data to identify nine genes associated with the prognostic response to NAC: *NDRG1*, *CXCL14*, *HOXB2*, *NAT1*, *EVL*, *FBP1*, *MAGED2*, *AR* and *CIRBP*. Furthermore, we constructed a prognostic risk model specifically linked to NAC. The clinical independence and generalizability of this model were effectively demonstrated. Additionally, we explore the underlying cancer hallmarks and microenvironment features of this NAC response-related risk score, and further assess the potential impact of risk score on drug response. In summary, our study constructed and validated a nine-gene signature associated with NAC prognosis, which was accomplished through the integration of single-cell and bulk RNA data. The results of our study are of crucial significance in the prediction of the efficacy of NAC in BC, and may have implications for the clinical management of this disease.

**Supplementary Information:**

The online version contains supplementary material available at 10.1186/s12920-023-01727-0.

## Background

Breast cancer (BC) is the most common cancer among women and a leading cause of cancer-related deaths worldwide, accounting for approximately 11.6% of all cancer deaths [1, 2]. Neoadjuvant chemotherapy (NAC) has emerged as the standard treatment for stage II – III BC in women, as the postoperative pathological complete response (pCR) status have been used for individualized systemic adjuvant treatment [3]. NAC can provide systemic chemotherapy for naive BC patients without metastasis before planned surgical treatment or local surgery plus radiotherapy, and additionally, and it is also the standard approach for locally advanced BC patients, enabling downstaging of inoperable tumors and facilitating breast-conserving surgery [4]. Moreover, NAC can obtain information related to drug sensitivity in vivo, guiding follow-up treatment and improve the prognosis of patients. Nonetheless, some patients may face significant challenges to their survival in cases where NAC proves ineffective. This is due to the fact that their prognosis may worsen, and they may experience severe toxic side effects [5]. Those patients who will not benefit from NAC will experience side effects of chemotherapy without any benefit, so we need biomarkers of NAC response to identify subgroups of patients who may benefit from NAC. Therefore, the identification of biomarkers predicting NAC is of great importance in treatment guidance based on NAC as well as other alternatives combination therapy [6].

Studies by Li J et al. revealed that the higher the level of aldehyde dehydrogenase 1 (ALDH1) in BC patients are associated with poorer response to NAC [7]. Wang et al. demonstrated that measuring the expression of matrix metalloproteinase-9 (MMP-9) in tumor tissues helps to identify TNBC patients who respond well to NAC [8]. In addition, targeting key molecules in signaling pathways, such as AKT/pERK and Fas/FasL, has shown potential in BC sensitivity to chemotherapy [8, 9]. However, most previous studies have mainly focused on individual biomarker, while integrating high-throughput multi-omics data can provide a more comprehensive understanding of the mechanisms underlying BC [10], reveal cellular heterogeneity and diversity, and identify biomarkers reflecting the complexity of these processes.

Genomic tests, such as Mammaprint and Oncotype DX, are used in the management of BC [11, 12]. Mammaprint examines the activity of 70 genes to categorize breast cancer as low or high risk of recurrence, providing a recurrence score indicating the likelihood of cancer returning. However, its validation primarily pertains to early-stage, ER+, LN-, and untreated patients. Mammaprint’s limitations include its limited applicability to other breast cancer subtypes, relatively high cost, and absence of direct treatment benefit information. Oncotype DX analyzes the expression of 21 genes and provides a recurrence score to predict distant recurrence and potential chemotherapy benefits in ER+, LN-, and HER2-negative breast cancers. However, it may not be suitable for HER2-positive or triple-negative breast cancer patients. Oncotype DX’s limitations include its limited application to certain breast cancer subtypes, challenges in interpreting intermediate recurrence scores, and concerns about cost and insurance coverage.

Currently, reliable biomarkers for predicting NAC in BC remain limited [13, 14]. Single-cell RNA sequencing (scRNA-seq) can contribute to identifying distinct cell populations involves in carcinogenesis and profiling marker genes at single-cell level [15, 16]. Understanding the heterogeneity of tumor microenvironment (TME) in drug resistance mechanisms and identify more effective targets for individualized management [17]. In this study, we aimed to identify potential NAC related prognostic signatures for predicting response to NAC in BC patients through integrated bioinformatics transcriptome analyses at both the single-cell and bulk levels.

## Methods

### Data collection and preprocessing

The bulk transcriptome data and corresponding clinical data of BC patients receiving NAC were obtained from the Gene Expression Omnibus (GEO) database (https://www.ncbi.nlm.nih.gov/geo/) via GSE25055, GSE25065 and GSE22226. We chose GSE25055 with the most BC samples (N = 306) as the discovery cohort, then 198 BC samples from GSE25065 and 150 BC samples from GSE22226 were chosen as independent validation cohorts. Samples with incomplete survival information were excluded from the analysis.

The scRNA-seq data from 14 BC samples was obtained from the study of Qian et al [18], and downloaded from lambrechtslab - Laboratory of Translational Genetics (vib.be). The R package Seurat (version 4.0.0) was used to preprocess the scRNA-seq. Cell samples with more than 200 genes expressed and the mitochondrial gene expression rate less than 5% were retained. We used the “NormalizedData” function to standardize the scRNA-seq dataset and the “FindVariableFeatures” function to identify 2000 highly variable genes. We used R package Harmony to correct the batch effects. After data normalization, the principal component analysis (PCA),was performed and cells were grouped and visualized using uniform manifold approximation and projection (UMAP) [19]. The “DotPlot” function was then used to visualize the expression level of marker genes in a single cluster [20]. These clusters are assigned to known cell lineages by marker genes. Clusters of cells are identified using the K-nearest neighbor (KNN) algorithm and the “FindClusters” function with a resolution of 0.2.

### Identification of candidate NAC marker genes and their cell expression activity

In the discovery cohort (GSE25055) with bulk transcriptome data, the differentially expressed genes (DEGs) between NAC resistant and sensitive groups were identified using the R package limma, with |log2FC |>0.585 and FDR < 0.05. For single-cell data, cell-specific genes were first identified using “FindAllMarkers” function in R package Seurat [18]. Then we obtained the intersection of NAC associated DEGs and cell-specific genes as the candidate NAC marker genes.

We used the R package AUCell (Version 1.12.0) to calculate the expression score of candidate NAC marker genes in each cell. The AUC value estimates the proportion of highly expressed genes in the gene set within each cell, and establishes a gene expression ranking for each cell. To calculate the threshold, we used the “AUCell_explore Thresholds” method.

### Construction of NAC prognosis model

Univariate Cox regression analysis was utilised to screen the prognostic value of candidate NAC marker genes. Then the least absolute shrink-age and selection operator (LASSO) Cox regression model were used to construct a prognostic model to minimize the risk of over-fitting and reduce the redudant factors [21]. LASSO algorithm selects and contracts variables through R packet glmnet.

The risk score of patients was calculated according to the expression level of each prognostic related gene and its corresponding regression coefficient:

risk score =$$\sum _{i=1}^{n}{{exp}_{i}}^{*}{\beta }_{i}^{}$$,

Where n is the number of prognostic genes, exp_i_ is the expression value of gene i, β_i_ is the regression coefficient of gene i. According to the median risk score, the patients were divided into high-risk and low-risk group.

### Assessment of the relevance with clinical variables

According to different clinicopathological characteristics, patients were divided into different subgroups, including age (> 50 and < = 50), grade (1/2 and grade 3/4), stage (I-II and III-IV), etc. Fisher’s exact test was used to compare the differences of each clinical variable between high- and low-risk groups, p < 0.05 was the significance threshold.

### Estimation of immune cell infiltration in the TME

The single sample gene set enrichment analysis (ssGSEA) algorithm was used to quantify the relative abundance of each cell infiltration in the TME of BC patients [22]. We obtained the signature gene sets indicating a wide range of human immune cell subtypes from Charoentong’s research, including activated CD8 T cells, activated dendritic cells, macrophages, natural killer T cells, and regulatory T cells [23]. Also, the R package ESTIMATE was used to calculate the immune score and ESTIMATE score.

### Chemotherapy sensitivity analysis

First, the IC50 values of drugs for each sample in the discovery cohort were calculated based on Genomics of Drug Sensitivity in Cancer (GDSC) (https://www.cancerrxgene.org) resource using calcPhenotype method from R package oncoPredict. To assess the correlation between small-molecule drug sensitivity and risk score, we calculated the Spearman correlation between risk score and drug IC50 values, and compared the differences of drug IC50 values between the high- and low-risk groups [24, 25].

### Statistical analysis

All statistical analyses were performed using R software (version 4.0.0; https://www.R-project.org). The difference of immune cell infiltration was assessed by Wilcoxon rank-sum test. For survival analysis, univariate and multivariate Cox analyses were used to explore the prognostic value and independence. R package survminer was used. Kaplan-Meier (KM) curves were plotted to visualize differences in overall survival (OS) between groups, and log-rank tests were used to assess the significance of these differences. Time-dependent Receiver Operating Characteristic (ROC) curve analysis was employed to evaluate the sensitivity and specificity of the risk score in predicting prognosis, and using the R package timeROC. Functional enrichment analysis was performed using the R package cluster Profiler.

## Result

### Single-cell analysis revealed microenvironment heterogeneity of BC

**Fig. 1:**
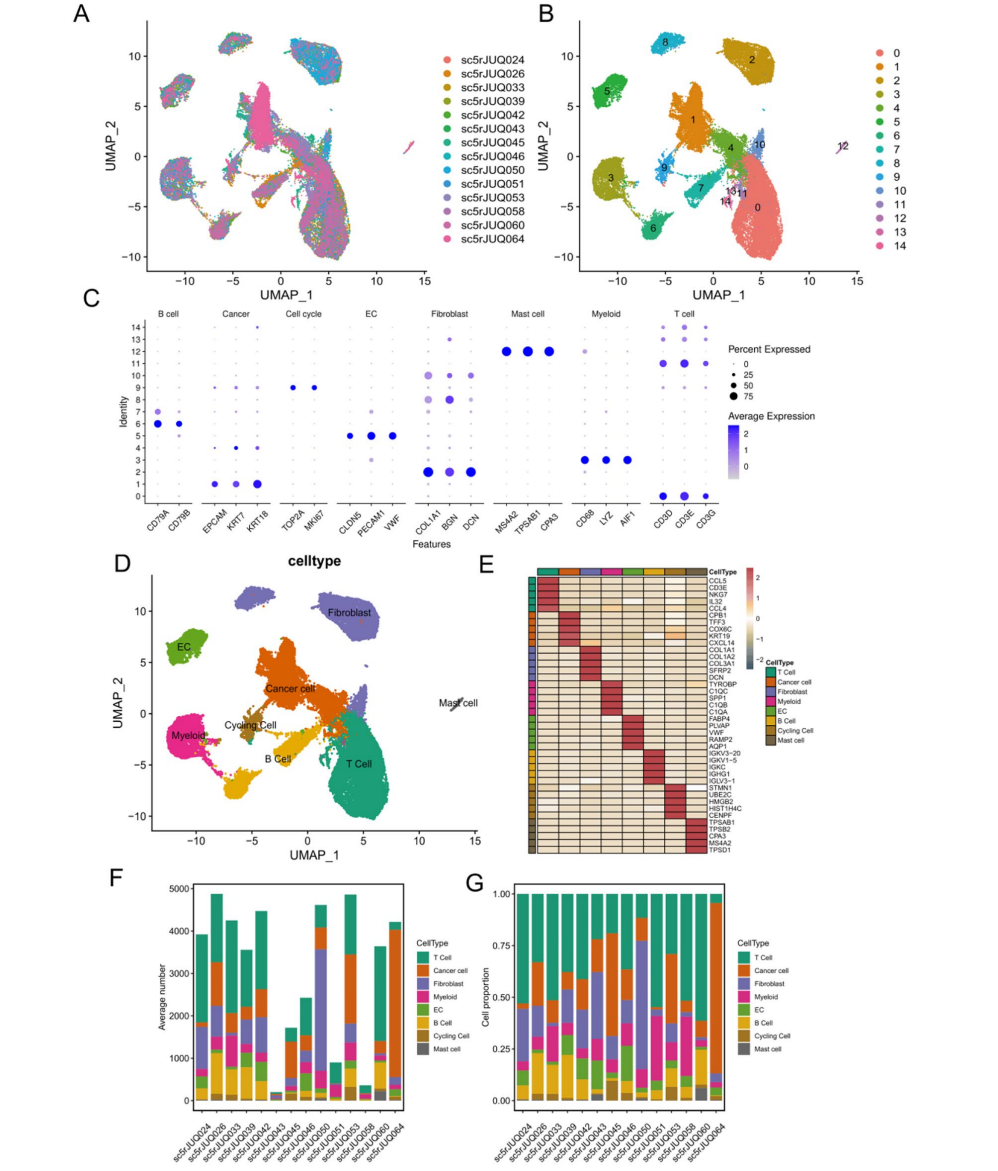
Analysis of cell subsets of single-cell RNA sequencing (scRNA-seq) from Breast Cancer (BC) patients. **A.** All the cell samples showed no significant batch effect; **B.** UMAP plot represents 15 cell clusters from 14 BC patients; **C.** The average expression of cell type marker genes in eight different cell types; **D.** UMAP plot represents the final identified eight cell types from (different colors represent different cell types) 14 BC patients; **E.** The expression of the top five highly expressed genes in each cell type; **F.** The barplot shows the total number of cell samples from each BC patient; **G.** The proportion of different cell types in each BC patient

Firstly, to investigate the heterogeneity of TME in BC patients, we obtained scRNA-seq data from 14 BC patients. After preprocessing and quality control, 44,024 cells were screened and UMAP analysis was performed to visualize the high-dimensional scRNA-seq data (Fig. 1A). Cell clustering revealed 15 subclusters (Fig. 1B), which were further annotated to 8 cell types based on marker genes expression (Fig. 1C, D). The top 5 highly expressed genes in each cell type were shown in Fig. 1E. It was worth noting that the cell composition of TME was highly heterogeneous, and the proportion of 8 cell types varied greatly among samples (Fig. 1F, G).

### Neoadjuvant chemotherapy signature identification through integrated single-cell and bulk RNA-seq data

**Fig. 2:**
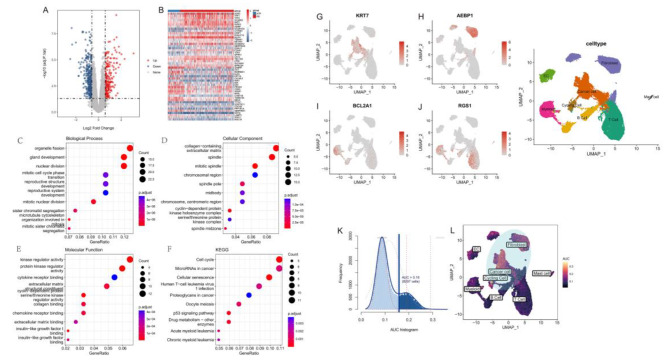
Identification of NAC-related differentially expressed genes and enrichment analysis. **A:** Volcano plot compares the differentially expressed genes (DEGs), screened by the criteria of |log2FC|>0.585 and p < 0.05, the blue dots denotes the down-regulated DEGs and the red dots denotes the up-regulated DEGs; **B:** Heatmap of DEGs indicating the expression of the top 50 DEGs in NAC resistant and sensitive groups, each row represents one DEG and each column represents one sample. The red and blue colors represent up-regulated and down-regulated DEGs respectively; **C:** GO-Biological process (BP) enrichment analysis results of NAC-related DEGs; **D:** GO-Cellular component (CC) enrichment analysis results of NAC-related DEGs; **E:** GO-Molecular function (MF) enrichment analysis results of NAC-related DEGs; **F:** Kyoto Encyclopedia of Genes and Genomes (KEGG) enrichment analysis results of NAC-related DEGs, the dot size represents the count of DEGs, and the color depth represents the p-value based significance; **G:** Expression of *KRT7* in different cell clusters; **H:** Expression of *AEBP1* in different cell clusters; **I:** Expression of *BCL2A1* in different cell clusters; **J:** Expression of *RGS1* in different cell clusters; **K:** AUC histogram for cell activity score of candidate NAC marker genes. The threshold was set as 0.16, and 9297 cells exceeded the threshold; **L:** The UMAP map is based on the candidate NAC marker genes score of each unit. Cell clusters with high ISG scores are highlighted

Due to the lack of single-cell data from BC patients received NAC, we first identified NAC response-related genes based on bulk RNA-seq. Transcriptome data and clinical information from 306 BC patients underwent NAC were obtained from the GEO database (GSE25055), of which 57 patiences achieved pCR and 249 patiences were therapy-resistant. Then, 551 DEGs between the NAC sensitive group and resistant group were identified based on the R package limma (Fig. 2A, |log2FC| > 0.585, FDR < 0.05). As shown in Fig. 2B, among the top 50 DEGs, *CA12* and *TFF3* were found to be significantly increased in BC patiences who often showed worse NAC efficacy. The high and correlated expression of *CA12* and *TFF3* in estrogen receptor-positive BC may play a role in reducing the tumor’s sensitivity to chemotherapy drugs such as adriamycin and docetaxel, which in turn can negatively impact the efficacy of NAC [26].

To further obtain NAC response-related genes that vary among different cell types, we identified190 intersecting key genes that were both NAC-response DEGs and highly variable genes of single-cell data. GO enrichment analysis revealed that these key genes are mainly distributed in the extracellular matrix and spindle and participate in the cell processes such as chromatid separation, cell cycle, mitosis, etc. By binding with chemokine receptors or regulating protein kinase (Fig. 2C-E). In addition, the results of KEGG [27–29] enrichment analysis showed that they were mainly involved in cell cycle, cellular senescence, and P53 signal pathways, among others (Fig. 2F).

**Fig. 3:**
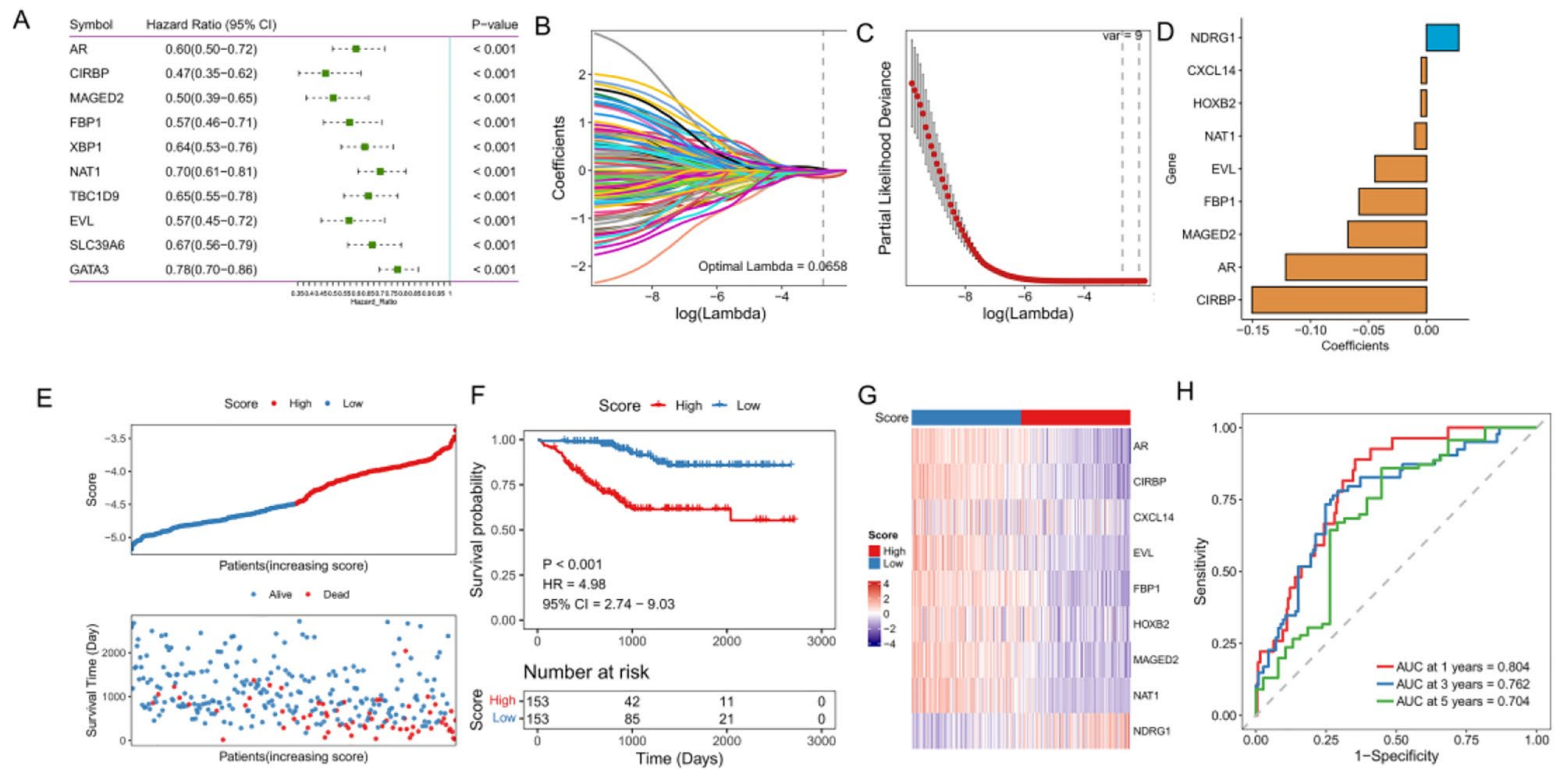
Construction and verification of NAC prognostic risk model. **A:** Forest map shows the results of the univariate analysis of the top 10 NAC-related key genes; **B:** LASSO coefficient profiles of the prognostic genes. Change track of each independent variable in LASSO Cox regression analysis. The horizontal axis represents the log value of the independent variable lambda, and the vertical axis represents the coefficient of the independent variable; **C:** Cross-validation for turning parameter selection in the LASSO regression model. Two vertical dashed lines indicated the optimal values using the minimum criteria. Optimal genes with the best discriminative capability were selected for developing the Prognostic model; **D:** LASSO regression coefficients of the 9 optimized genes for constructing the prognostic model; **E:** Risk score distribution for each sample in the GSE25055 cohort; **F:** Kaplan-Meier curves comparing the OS of patients separated by risk groups, the red line represents line the high-risk score group, while the blue line represents the low-risk score group; **G:** Expression of the 9 prognostic model genes in the GSE25055 cohort between high- and low-risk group; **H:** Time-dependent receiver operating characteristic (ROC) curve of the prognostic model

In addition, we observed the cell-specific expression pattern of the 190 key genes of NAC response. Among them, *KRT7* was highly expressed in cancer cells, *AEBP1* was mainly expressed in fibroblasts, *BCL2A1* and *RGS1* were both highly expressed in myeloid cells (Fig. 2G-J). Specifically, *RGS1* was also specifically expressed in T cell clusters, and a previous study has supported that *RGS1* was associated with CD4 expression and was functionally associated with T cell activation [30]. The expression activity of the 190 key genes was calculated using the R package AUCell and showed a bimodal distribution among all cells (Fig. 2K). According to the bimodal distribution threshold of 0.16, all cells were divided into two groups of high and low activity. The results showed that 9297 cells (9297/44,024, 21%) had higher expression activity of NAC response-related genes, mainly including fibroblasts, cancer cells, and cycling cells (Fig. 2L). Further enrichment analysis of cell-specific genes showed that their functions were mainly related to oxidative phosphorylation, energy metabolism and DNA replication (Fig. S1).

### Construction and validation of NAC prognostic model

To assess the prognostic value of key NAC response-related genes in patients with BC, univariate Cox analysis and log-rank test was performed in 306 BC patients underwent NAC (GSE25055) for 190 genes. The results showed that 126 genes were associated with OS in BC (P < 0.05), and the top 10 genes with the most significant p values were shown in Fig. 3A and S2. Among them, *GATA3* is a key transcription factor involved in the development of breast tumors. Here, we found the higher expression of *GATA3* indicated a better prognosis, which was supported by a previous analysis that *GATA3* was required for homologous recombination repair and served as a tumor suppressor [31].

In order to construct a promising prognostic model based on the NAC response-related genes, we performed LASSO-Cox analysis to remove redundant prognostic factors (Fig. 3B, C), the resulting prognostic model, when applied to patients, can effectively predict OS outcomes. And nine prognostic genes related to NAC response were retained to construct the model, including *NDRG1*, *CXCL14*, *HOXB2*, *NAT1*, *EVL*, *FBP1*, *MAGED2*, *AR* and *CIRBP* (Fig. 3D). According to their expression levels, we measured the prognostic risk score for each patient and separated them into two groups based on the median score (Fig. 3E). K-Mcurve with the log-rank test further showed a significant reduction in OS in the high-risk score group (log-rank test p-value < 0.001, Fig. 3F). The nine prognostic genes related to NAC response had distinct expression patterns in different risk groups. *NDRG1* was the only gene that up-regulated in the high-risk group, while the other eight genes were up-regulated in the low-risk group (Fig. 3G). ROC curve analysis was used to evaluate the prediction efficiency of the NAC prognostic model. And the area under the ROC curve (AUC) reached 0.804, 0.762 and 0.704 at 1, 3 and 5 years, respectively, indicating that the prediction effect of the model is reliable (Fig. 3H).

**Fig. 4:**
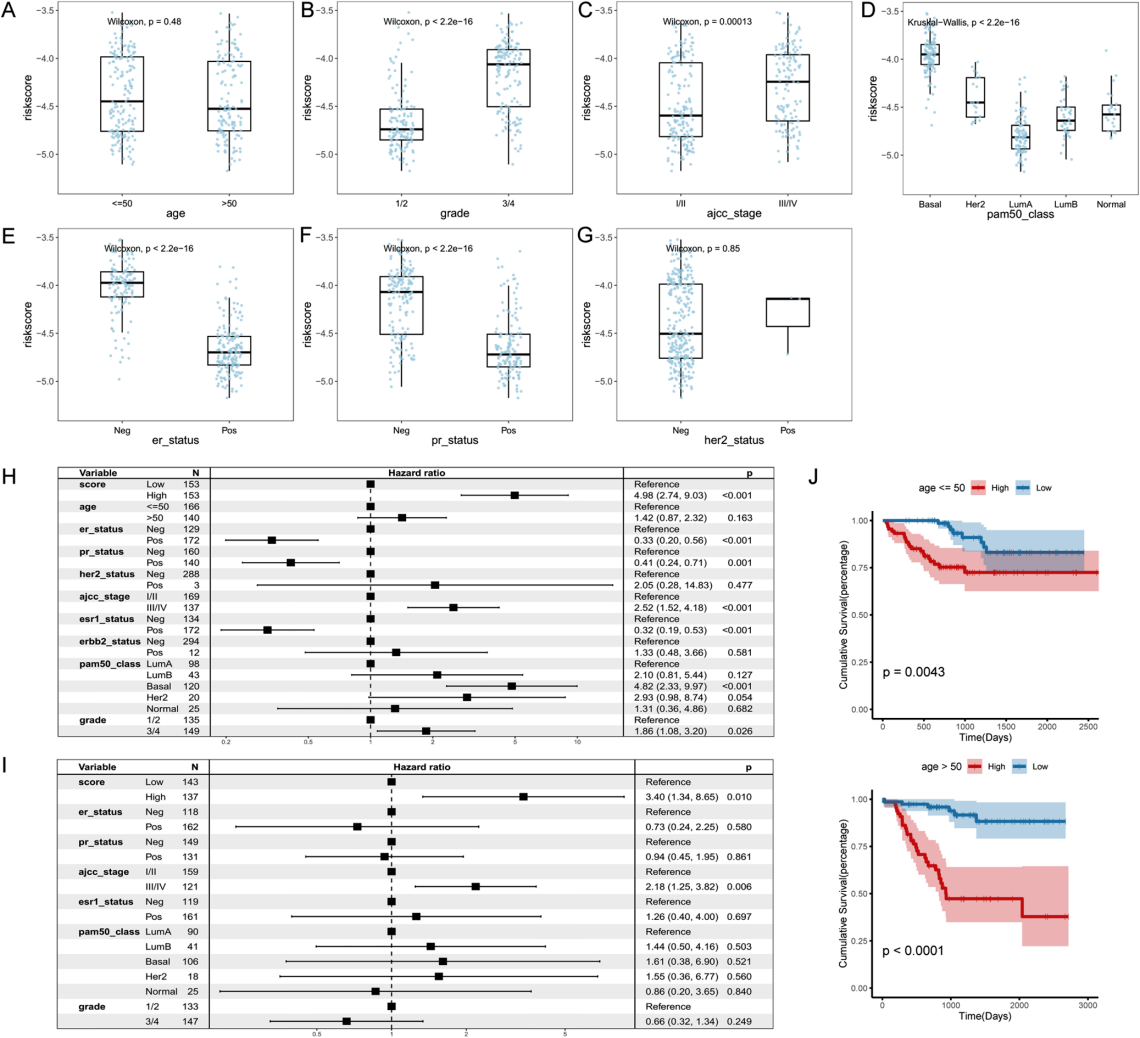
Clinicopathological significance of NAC response-related risk score in clinical variables.**A:** Differences in NAC response-related risk scores between age group; **B:** Differences in NAC response-related risk scores across different tumor grade; **C:** Differences in NAC response-related risk scores within different TNM stage; **D:** Differences in NAC response-related risk scores in different intrinsic subtypes; **E:** Differences in NAC response-related risk scores across ER status; **F:** Differences in NAC response-related risk scores across PR status; **G:** Differences in NAC response-related risk scores across HER2 status; **H:** Univariate cox analysis of risk score in the GSE25055 cohort; **I:** Multivariate cox analysis of risk score in the GSE25055 cohort; **J:** K-M curve of NAC prognosis model in different groups of age

In addition, we assessed the robustness and generalizability of the NAC prognostic model in two independent validation cohorts. Consistently, we calculated the risk score for each patient and separated them as the discovery cohort did. As expected, the high-risk group showed significantly reduced OS compared to the low-risk group (log-rank p-value < 0.001 for GSE25065 and GSE22226, respectively). It’s worth noting that the prognostic model also showed good predictive performance in external validation sets with the predictive AUC reached 0.87 and 0.753 respectively (Fig. S3).

### Association of NAC risk scores with clinical variables

To further investigate the clinicopathologic significance of the NAC response-related risk score, we separated 306 patients by clinicopathologic variables and compared the difference in risk scores between groups. As expected, the risk score was significantly associated with tumor grade and TNM stage, indicating that as the disease progresses, the NAC response-related risk score increases (Wilcoxon rank-sum test p-value < 0.001, Fig. 4A, B). However, we didn’t observe its association with age (Fig. 4C). As for the three indicators of triple-negative breast cancer (TNBC), the risk score was significantly higher in ER and PR-negative groups (Wilcoxon rank-sum test p-value < 2.2e-16, Fig. 4D, E), while it didn’t show a relationship with Her-2 status, probably due to the limited sample size of Her-2^+^ patients (Fig. 4F). Simultaneously, among the intrinsic subtypes (PAM50) of BC, the basal-like (also considered as TNBC) subtype showed the highest risk score compared other groups (Fig. 4G). The two independent validation cohorts (GSE25065 and GSE22226) supported the clinicopathologic associations of NAC response-related risk model (Fig. S4). These results suggested that the NAC response-related risk score reflected a worse prognosis of TNBC [32].

In addition, we investigated the independence and predictive efficiency of the NAC response-related risk score in the 306 BC patients underwent NAC (GSE25055). Univariate Cox regression analysis revealed the prognosis associations (with OS) of the risk score, ER and PR status, AJCC stage, and tumor grade (HR = 4.98, 95% CI = 2.74–9.03, P < 0.001, Fig. 4H). Then, the multivariate Cox regression analysis showed that the risk score remained an independent predictor of OS after adjusting for other confounding factors(HR = 3.40, 95% CI = 1.34–8.65, P = 0.01, Fig. 4I). These results were also found in the two independent validation cohorts (Fig. S5A, B). To explore the applicability of the NAC prognostic model, we compared its prognosis associations in different clinical features groups. In particular, there was a strong prognostic effect of the NAC prognostic model regardless of whether the risk score was associated with this clinical variable (log-rank P < 0.05, Fig. 4J, S5C-F).

### NAC risk score characterized differential tumor hallmarks and microenvironment features

**Fig. 5:**
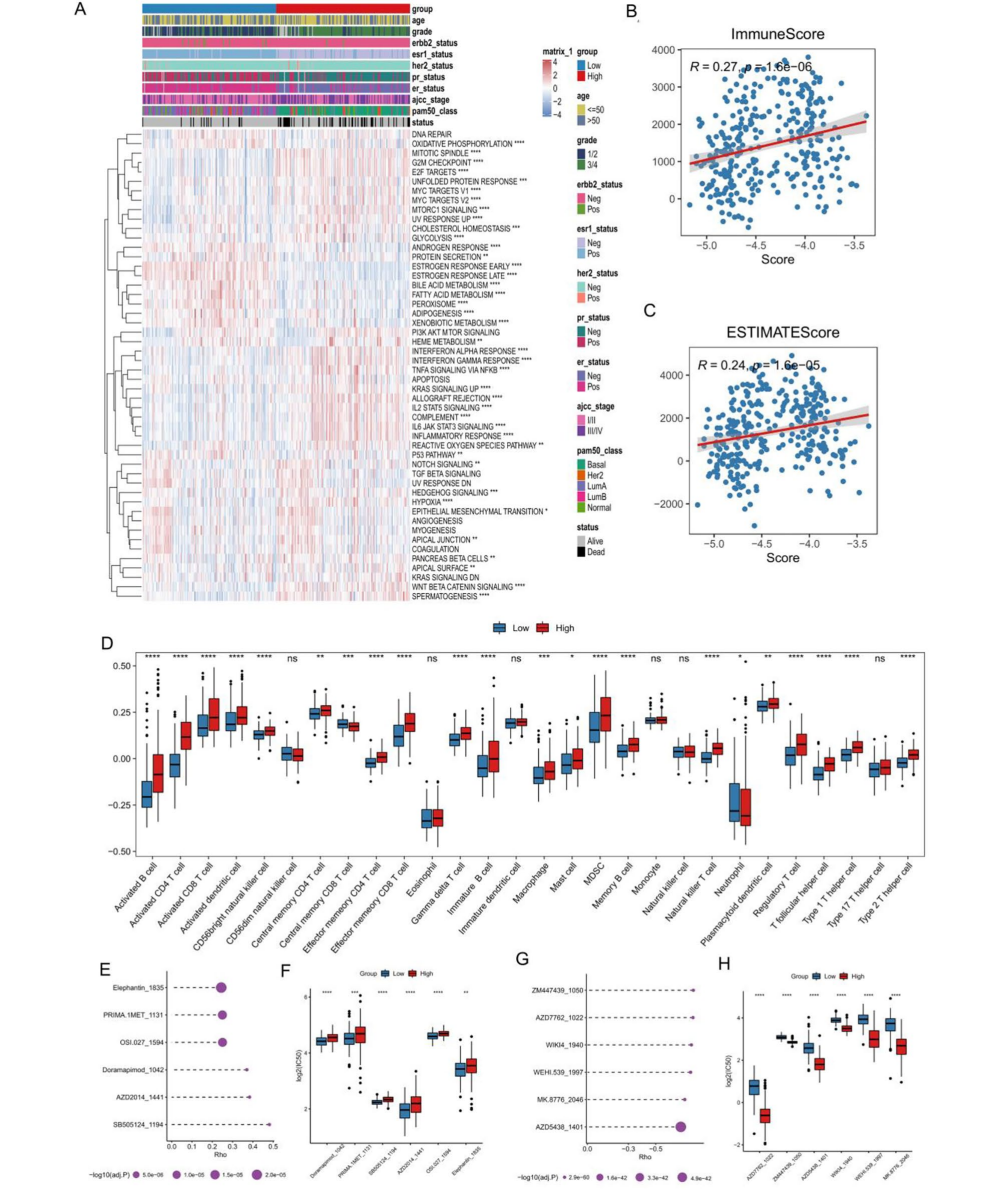
The correlation between NAC risk score of patients and characterization of the immune microenvironment. **A:** Heat plot of the activity of cancer hallmarks in the GSE25055 cohort between high- and low-risk group; **B:** Spearman correlation between the NAC response-related risk score and the immune score (R = 0.27; p < 0.01); **C:** Spearman correlation between the NAC response-related risk score and the ESTIMATE score; **D:** The proportions of differentially infiltrated TME immune cells between the high- and low-risk score groups; **E:** Top 6 drugs with the highest positive correlation with risk score; **F:** Comparison of log2 (IC50) values of the top 6 positively correlated drugs between the high- and low-risk score groups; **G:** Top 6 drugs with the highest negative correlation with risk score; **H:** Comparison of log2 (IC50) values of the top 6 negatively correlated drugs between the high- and low-risk score groups

In order to explore the underlying molecular mechanisms of this NAC response -related risk score, we first investigated the cancer hallmarks associated with the risk score. We calculated the activity score of 50 cancer hallmark pathways for 306 BC patients underwent NAC. The results indicated that 41 of the 50 pathways had significant differences between the high- and low- risk groups (Fig. 5A). For instance, the interferon-α/γ response, inflammatory response, Wnt-β catenin signaling, glycolysis, apoptosis, KRAS signaling, and hypoxia hallmarks were significantly activated in the high-risk group, whereas DNA repair, oxidative phosphorylation, and fatty acid metabolism hallmarks were significantly activated in the low-risk group.

As the risk score showed a relationship with inflammatory response, we further explored the association between the risk score with immune response. Using the ESTIMATE algorithm, we found that the NAC response-related risk score was slightly positively correlated with immune and ESTIMATE score of the TME (Spearman correlation analysis, Fig. 5B, C). Additionally, we analyzed the difference in immune cell infiltration between the high- and the low- risk groups. To our surprise, activated B cell, CD4 T cell, CD8 T cell, dendritic cell and effector memory CD8 T cell was significantly accumulated in the high-risk group (Fig. 5D). Furthermore, the immune-suppressive cell types including MDSC and Regulatory T cell were increased in the high-risk group. Previous studies have shown that tumors with immune-excluded phenotypes which associated with immune cells embedded in the surrounding tumor stroma away from tumor cells, also have higher immune infiltration, but whether the effector cells were surrounding the tumor or suppressed by the microenvironment is unclear [33].

### NAC risk score and chemotherapy drug sensitivity

To further assess the potential impact of the risk score on drug response, we investigated drug response and potential therapeutic compounds based on the NAC response-related risk score. The spearman correlation between IC50 values and the risk score revealed the top 6 positive and top 6 negative correlated drugs (Fig. 5E, G). For the positively correlated drugs, the IC50 of Doramapimod, Elephantin, AZD2014, SB505124 and PRIMA-1MET differed significantly between the high and low groups (Fig. 5F). For negatively related drugs, there also was a substantial difference between the high- and low-risk groups (Fig. 5H). These results demonstrated that the risk score can be applied to drug resistance analysis.

## Discussion

BC has the highest incidence rate among female malignant tumors, accounting for 25% of the total incidence rate [34]. NAC is a treatment strategy that involves administering chemotherapy before surgery. Its goal is to reduce tumor volume and aggressiveness, leading to improved surgical resection rates and therapeutic effects. In the case of BC, NAC offers multiple benefits [35, 36]. However, the lack of effective prognostic biomarkers for NAC presents a significant challenge to its clinical application in BC [37]. To address this challenge, we conducted a characterization of 190 key prognostic genes related to NAC. This characterization was achieved by integrating bulk RNA-seq data from 306 BC patients (including 57 with pathologic complete response and 249 resistant cases) and single-cell RNA-seq data from 14 BC patients. Through LASSO-Cox analysis, we identified nine NAC-related prognostic signatures. Our study then encompassed a comprehensive analysis of both the training cohort, GSE205055, and the validation cohorts, GSE25065 and GSE22226. This analysis enabled us to establish and validate, for the first time, a prognostic risk model specific to NAC response. Univariate and multivariate Cox regression analyses further confirmed that this prognostic risk model represents an independent risk factor associated with OS. Additionally, we evaluated the prognostic model’s association with various clinical variables. The establishment of this NAC response-related prognostic risk score will empower clinicians to make more informed decisions regarding personalized treatment strategies and follow-up care. Ultimately, this model has the potential to significantly improve survival rates and treatment outcomes for patients with BC.

The pathogenesis of BC is a complex process influenced by various genetic and environmental factors [38]. When integrating bulk-RNA seq and scRNA-Seq screening signatures, the key difference from other methods like whole-genome sequencing (WGS) or whole-exome sequencing (WES) is that it focuses solely on gene expression rather than genomic variations or mutations. While WGS and WES provide valuable insights into DNA sequence variations, they do not directly capture gene expression profiles, making it difficult to infer functional differences at the transcriptional level. By combining bulk-RNA seq and scRNA-Seq screening signatures, we can unravel the underlying regulatory mechanisms and gain a deeper understanding of how specific genes and pathways contribute to the observed cellular heterogeneity.Through our research, we have gained insights into the potential roles of NAC-related genes in regulating multiple cellular processes associated with BC development and progression. Our KEGG enrichment analysis further indicates that the differentially expressed genes identified in our study may be involved in regulating biological processes, such as cellular aging and the P53 signaling pathway. These pathways have been implicated in various types of cancer, suggesting that the differentially expressed genes identified in our study could play crucial roles in BC pathogenesis [39–41]. Notably, the observation of high expression of NAC-related genes in fibroblast, cancer cell, and cycling cell types is particularly significant, as these cell types are known to have important roles in BC pathogenesis [42–45].

Moreover, our scRNA-seq analysis revealed a highly heterogeneous cell composition within TME of BC, with significant variations in the proportions of eight distinct cell types across samples. Notably, sample sc5Rjuq064 exhibited a decreased proportion of T cells and an increased proportion of tumor cells compared to other samples, which correlated with the tumor stage. Through enrichment analysis, we found that the differentially expressed genes in these three active cell types primarily relate to oxidative phosphorylation, energy metabolism, and DNA replication. Interestingly, contrary to previous research, cancer-related fibroblasts appear to act as direct positive regulators of the adaptive immune system, suggesting the potential use of immune-stimulating CAF in cancer treatment [46]. Zheng et al.‘s research implies that combining chemotherapy with anti-cancer-related fibroblast therapy may enhance the effectiveness of T cell-based immunotherapy, providing a potential strategy for colon cancer treatment [47]. The role of fibroblasts in BC warrants further investigation. Overall, our research enhances the understanding of BC pathogenesis by shedding light on the specific genes and cellular processes involved in its development and progression. These findings have the potential to contribute to the development of novel diagnostic and therapeutic approaches for BC.

In this study, LASSO-Cox regression analysis identified nine prognostic genes associated with NAC response: NDRG1, CXCL14, HOXB2, NAT1, EVL, FBP1, MAGED2, AR, and CIRBP. Among these genes, AR has been found to stimulate breast tumor growth in the absence of the estrogen receptor, making it a promising molecular target in the treatment of TNBC [48]. Dong et al. designed a novel combination therapy using enzalutamide and ceritinib to target both androgen-dependent and androgen-independent AR signaling pathways in TNBC tumors [49]. CIRBP, known for its ability to bind and post-transcriptionally regulate mRNA, has been linked to cancer promotion and inflammation [50, 51]. Recent studies have identified CST3 as a downstream mediator for CIRBP functionality [52]. The melanoma-associated antigen (MAGE) family proteins are recognized tumor-specific antigens. MAGED2 exhibits distinct effects depending on the subtype of breast cancer. It has been identified as a potential prognostic factor for wild-type TP53 patients and breast cancer patients with varying pathological grades [53].

Our research also investigated the potential utility of the NAC response-related risk score in predicting drug response to chemotherapy in breast cancer (BC). SB505124, a small molecule inhibitor of TGF-β receptor I (ALK5), was examined in this context. We observed that BC patients in the low NAC risk group exhibited significantly lower IC50 values for SB505124 compared to those in the high NAC risk group. This suggests that patients in the low NAC risk group may be more responsive to SB505124 treatment. Notably, in conjunction with SMAD3, SB505124 can attenuate the activity of CD8 + T cells, which may contribute to the reduced efficacy of immunotherapy in malignant BC [54]. These findings emphasize the potential of risk scoring as a valuable tool for predicting drug response and optimizing therapy in BC patients, ultimately leading to more effective and personalized treatment approaches. However, the clinical classification of breast cancer has a great influence on the treatment effect of NAC. Although we validated the applicability of our model in different clinical classification of breast cancer, further studies are needed.

In conclusion, our study integrated single-cell and bulk RNA sequencing analyses to construct and validate a nine-gene signature associated with NAC prognosis. This signature serves as an independent prognostic indicator for BC patients. Additionally, our findings provide genomic evidence for future research directions in developing anti-BC treatment strategies, particularly for individuals who may not benefit from NAC.

### Electronic supplementary material

Below is the link to the electronic supplementary material.

Supplementary Material 1

Supplementary Material 2

Supplementary Material 3

## Data Availability

The datasets GSE25055, GSE25065 and GSE22226 supporting the findings of this study are available in the from GEO (https://www.ncbi.nlm.nih.gov/geo/). For the discovery cohort databases. Further inquiries can be directed to the corresponding author.
