# Supplementary material for: Integrated single-cell and bulk RNA sequencing analysis identifies a neoadjuvant chemotherapy-related gene signature for predicting survival and therapy in breast cancer
Source: BMC Med Genomics. 2023 Nov 23;16:300. doi: 10.1186/s12920-023-01727-0 (PMC10666338; doi:10.1186/s12920-023-01727-0)
Supplement: Supplementary file 1 — Supplementary Material 1 [file 12920_2023_1727_MOESM1_ESM.docx]

**Fig. S1: BP Enrichment of Cancer Cell, Cycling Cell, and Fibroblast**


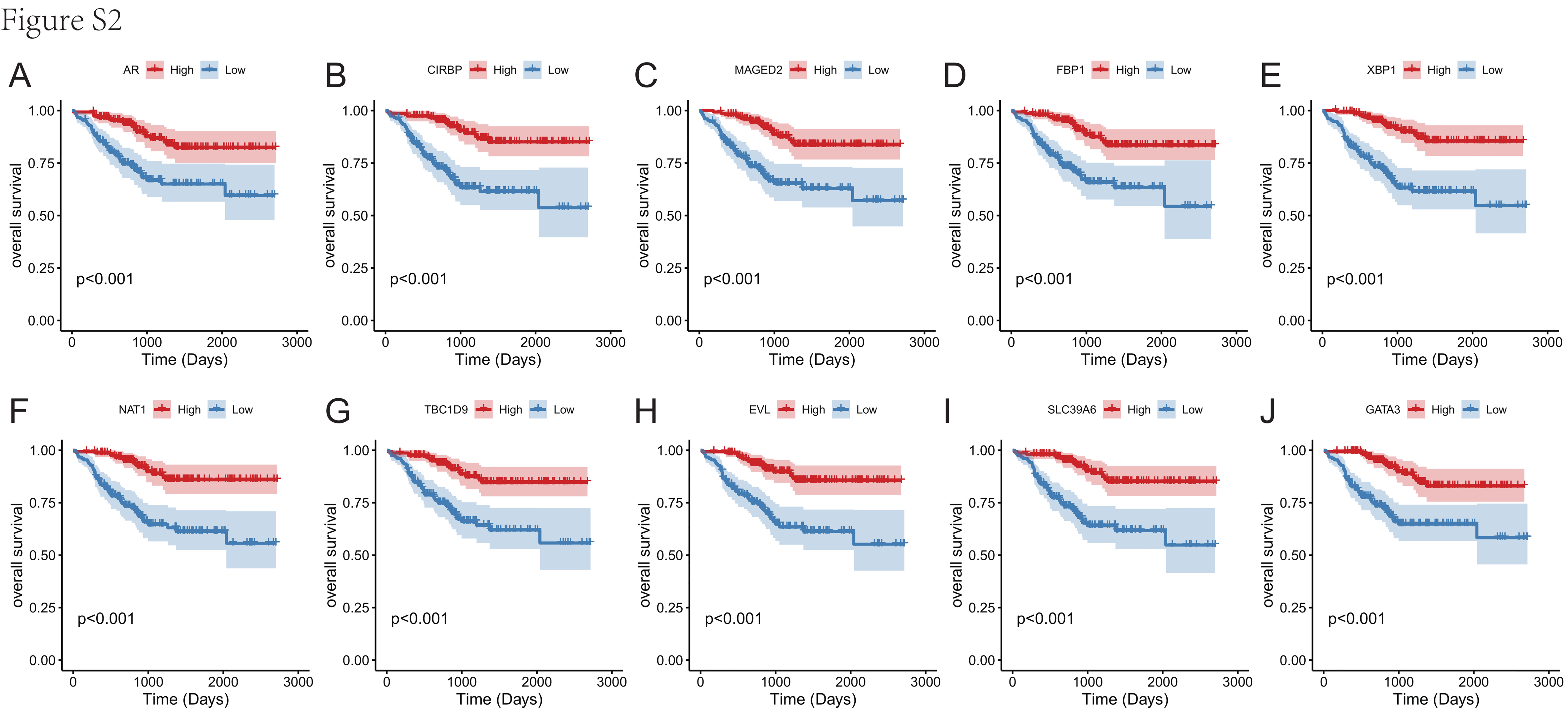


**Fig. S2 KM Curve of Top 10 NAC-related Key Genes**


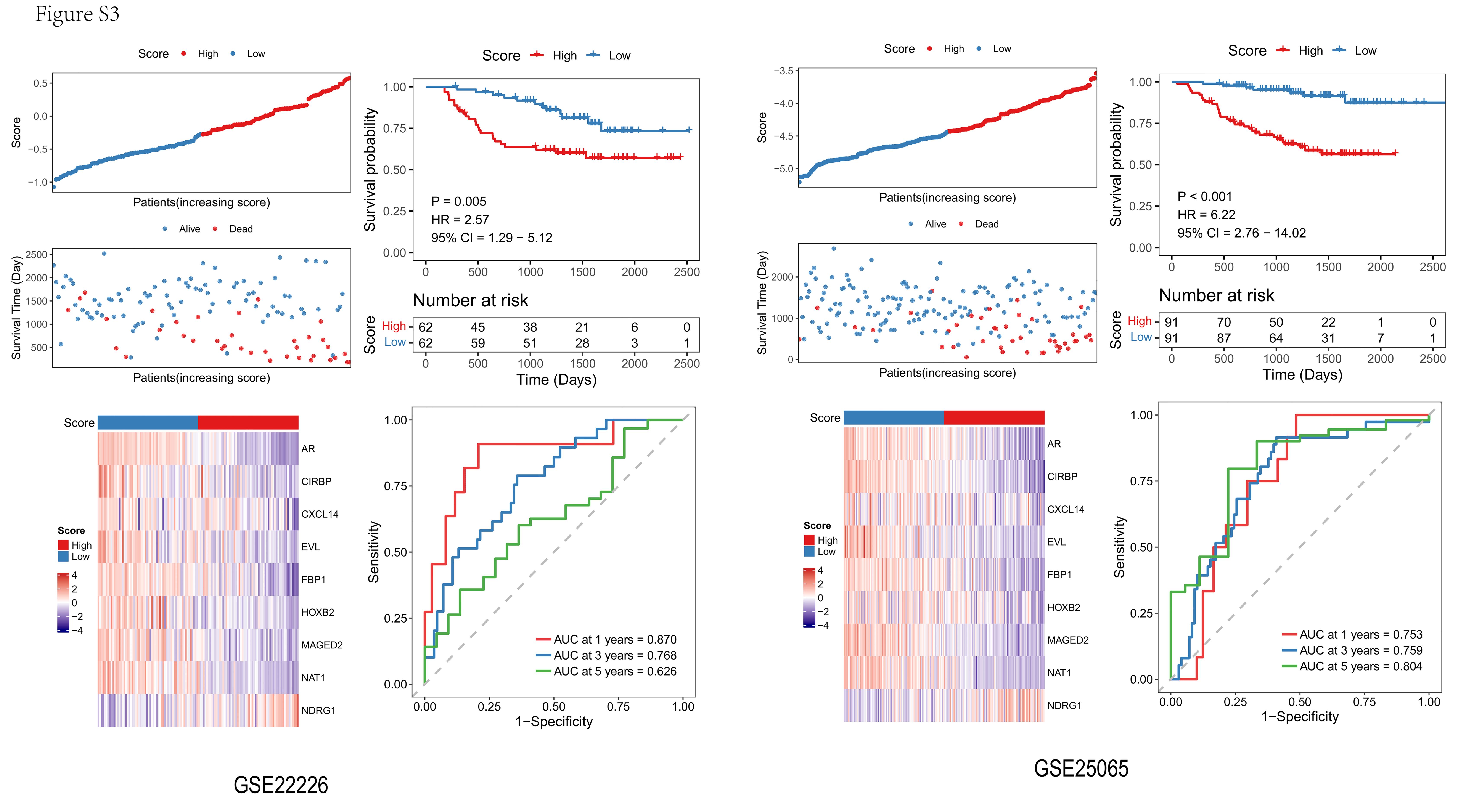


**Fig. S3 Validation of Prognostic Model in Validation Cohorts**


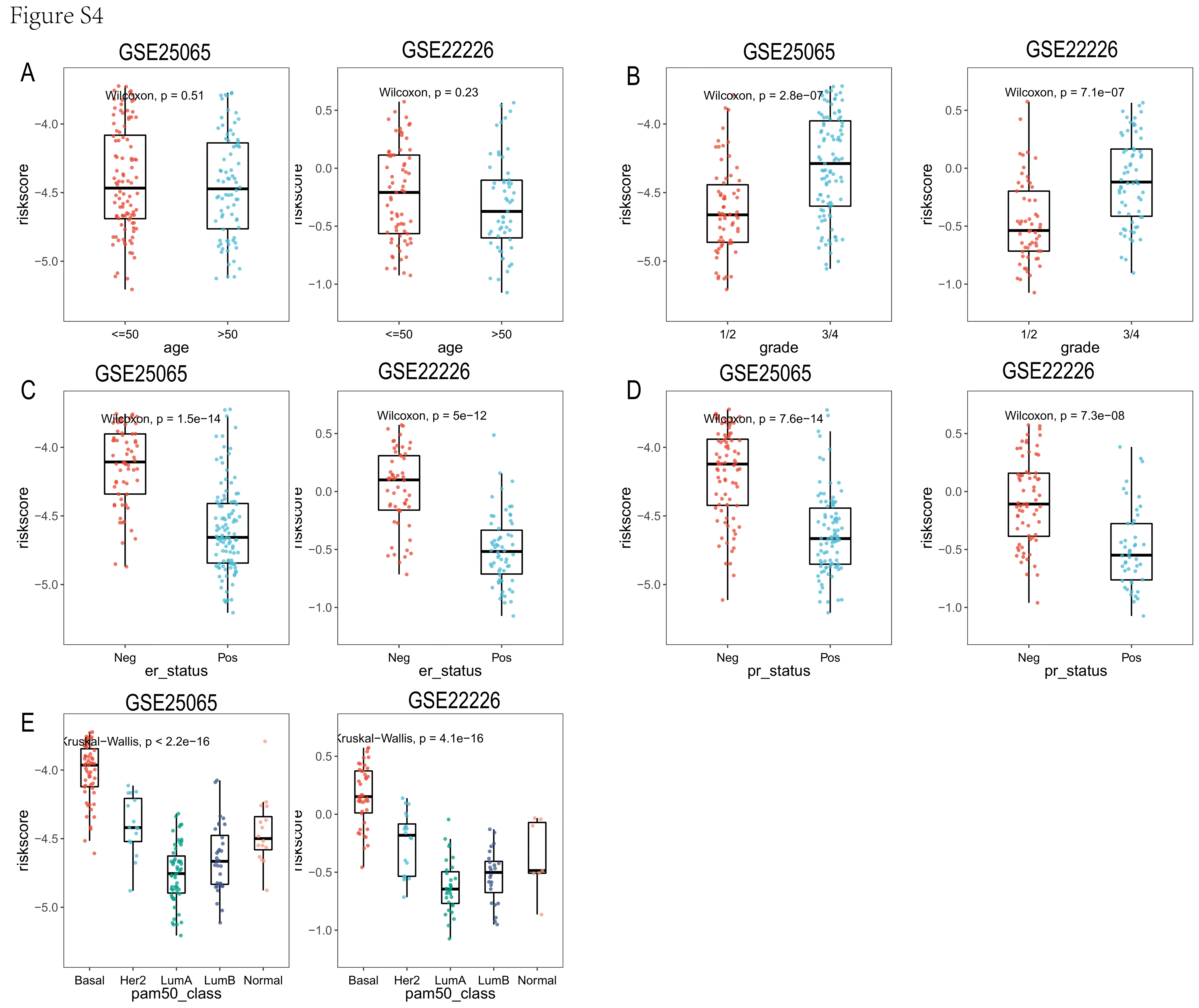


**Fig. S4** **Differences in Risk Scores Across Clinical Variable in the GSE25065 and GSE2226 Cohorts**

1. Differences in risk scores across age;
2. Differences in risk scores across tumor grade;
3. Differences in risk scores across ER status;
4. Differences in risk scores across PR status;
5. Differences in risk scores across the intrinsic subtypes;


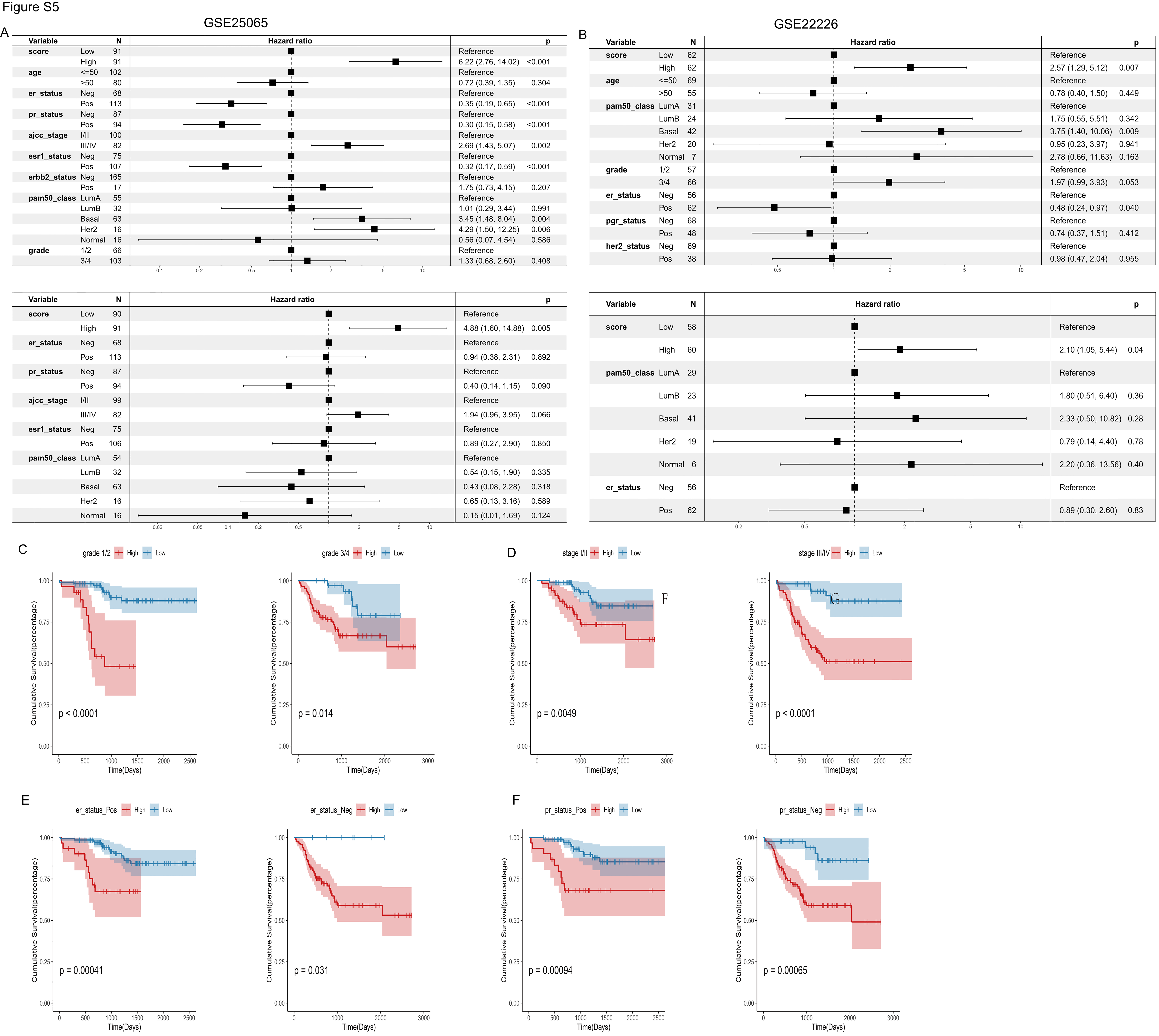


**Fig. S5** **Differences in Risk Scores Across Clinical Variable in the GSE25065 and GSE2226 Cohorts**

1. Univariate Cox (upper) and multivariate Cox (lower) analysis in the GSE25055 cohort;
2. Univariate Cox (upper) and multivariate Cox (lower) analysis in the GSE22226 cohort;
3. KM curve of NAC prognosis model related to grade 1/2；
4. KM curve of NAC prognosis model related to grade 3/4；
5. KM curve of NAC prognosis model related to stage Ⅰ/Ⅱ；
6. KM curve of NAC prognosis model related to grade Ⅲ/Ⅳ；
7. KM curve of NAC prognosis model related to ER-positive；
8. KM curve of NAC prognosis model related to ER-negative；
9. KM curve of NAC prognosis model related to PR-positive；
10. KM curve of NAC prognosis model related to PR-negative.
